# Density Functional Theory based study on structural, vibrational and NMR properties of *cis - trans* fulleropyrrolidine mono-adducts

**DOI:** 10.1371/journal.pone.0207635

**Published:** 2018-11-19

**Authors:** Seif Bennia, Rim Milad, Sabri Messaoudi, Marine de Person, Fathi Moussa, Manef Abderrabba, Denis Merlet

**Affiliations:** 1 Laboratoire Matériaux Molécules et Applications, IPEST, Université de Carthage, Route Sidi Bou Said, La Marsa, Tunisie; 2 Faculté des Sciences de Bizerte, Université de Carthage, Bizerte, Tunisie; 3 Equipe RMN en Milieux Orienté (ERMN) ICMMO—UMR 8182—Bât, Université Paris-Sud Université Paris) France; 4 LETIAM, Lip(Sys)^2^ EA, Université Paris Sud Université Paris Saclay, IUT d’Orsay Plateau de Moulon, Orsay, France; Aligarh Muslim University, INDIA

## Abstract

Since the early nineties, countless publications have been devoted to the study of possible uses of [60] fullerene (C_60_) and its derivatives in the fields of materials and nano-biomedical sciences. However, in spite of the importance of conformers notably from the pharmacological point of view, the cis/trans isomerization of C_60_ mono-adducts has been rarely seldom investigated. Here we present the results of DFT calculations of the structural, vibrational and NMR properties of both *cis* and *trans* isomers of fulleropyrrolidine mono-adduct obtained by photo-addition of glycine methyl ester to C_60_. Taken together, our results have shown that the *cis* isomer is more stable than the *trans* one. For the *cis* conformation, the simulated vibrational spectrum shows a more intense peak at 1298 cm^-1^. While ^13^C spectra revealed no significant differences between the two isomers as compared to experimental results, the calculated ^1^H chemical shifts show a significant difference between the two conformers in both the gas phase and in solution. The *trans* isomer presents a proton at 5.86 ppm, which is more deshielded than the proton of the cis conformer (5.24 ppm).

## Introduction

More than 30 years after its discovery, [60] fullerene or C_60_ and its derivatives have continueed to fuel research in the fields of materials and biomedical sciences [1,2]. Moreover, although the chemical reactivity of C_60_ has been well apprehended for years, some important chemical reactions have never been carried out on the surface of the fullerene [3]. Indeed, chemists still continue to exploring alternative ways of C_60_ derivatization [4].

Several important aspects of C_60_-derivatives characterization still remain to be investigated. This is true not only for poly-adducts and their isomers but also for simple mono-adducts. For instance, while the mechanism of sequential photo-addition of glycine methyl ester (GME) to C_60_ has been well understood [5,6,7], the separation and characterization of the whole of the obtained fulleropyrrolidine poly-adducts remain to be achieved. It is the same for the separation and the characterization of the *cis-trans* isomers of the fulleropyrrolidine mono-adduct [8]. This is an important issue notably because of the difference of reactivity of such a kind of conformers at the pharmacological level.

As a matter of fact, the *cis/trans* isomerisation of C_60_ derivatives has been rarely investigated. Only few studies have been devoted to this aspect of fullerene chemistry [9, 10,11]. More recently, based on experimental results and DFT calculations a mechanism of stereo-chemical outcome of *cis-trans* isomerization involving the H-bonding assistance of the inner water molecule in the carbanion stabilization of H20@C_60_ fulleropyrrolidine has been proposed [12].

Here we present the results of a density functional theoretical study on the structural, vibrational and NMR properties of both *cis* and *trans* isomers of the fulleropyrrolidine mono-adduct obtained by photo-addition of GME to C_60_ [5]. The obtained results are also compared to the previously published experimental results [5].

## Methodology

Gaussian 09 has been employed for all the theoretical calculations [13]. Geometries of structures were optimized using the DFT at B3LYP [14,15] level along with 6-31G (d) basis set [16–19]. The 6-31G (d) basis set [20,21] was chosen to provide accurate results for conjugated derivatives [22]. The Geometry optimizations and frequencies were performed in the gas phase. Frequencies were scaled by a factor amounting to 0.96 to best compare with experiment [23]. The calculation of NMR spectrum of the different structures was performed using the GIAO (Gauge-Including Atomic Orbitals) method [24, 25], implemented in the Gaussian, with the B3LYP functional, in conjunction with 6-31G(d) and 6–311+g(2d,p) basis sets. In order to express the chemical shifts in ppm, the geometry of the tetramethysilane (TMS) molecule has been optimized. The calculations of NMR spectrum were performed in the presence of CDCl_3_ solvent with the PCM model and in the gaseous phase.

## Results and discussion

### Structures of GME added to C_60_

**Fig 1:**
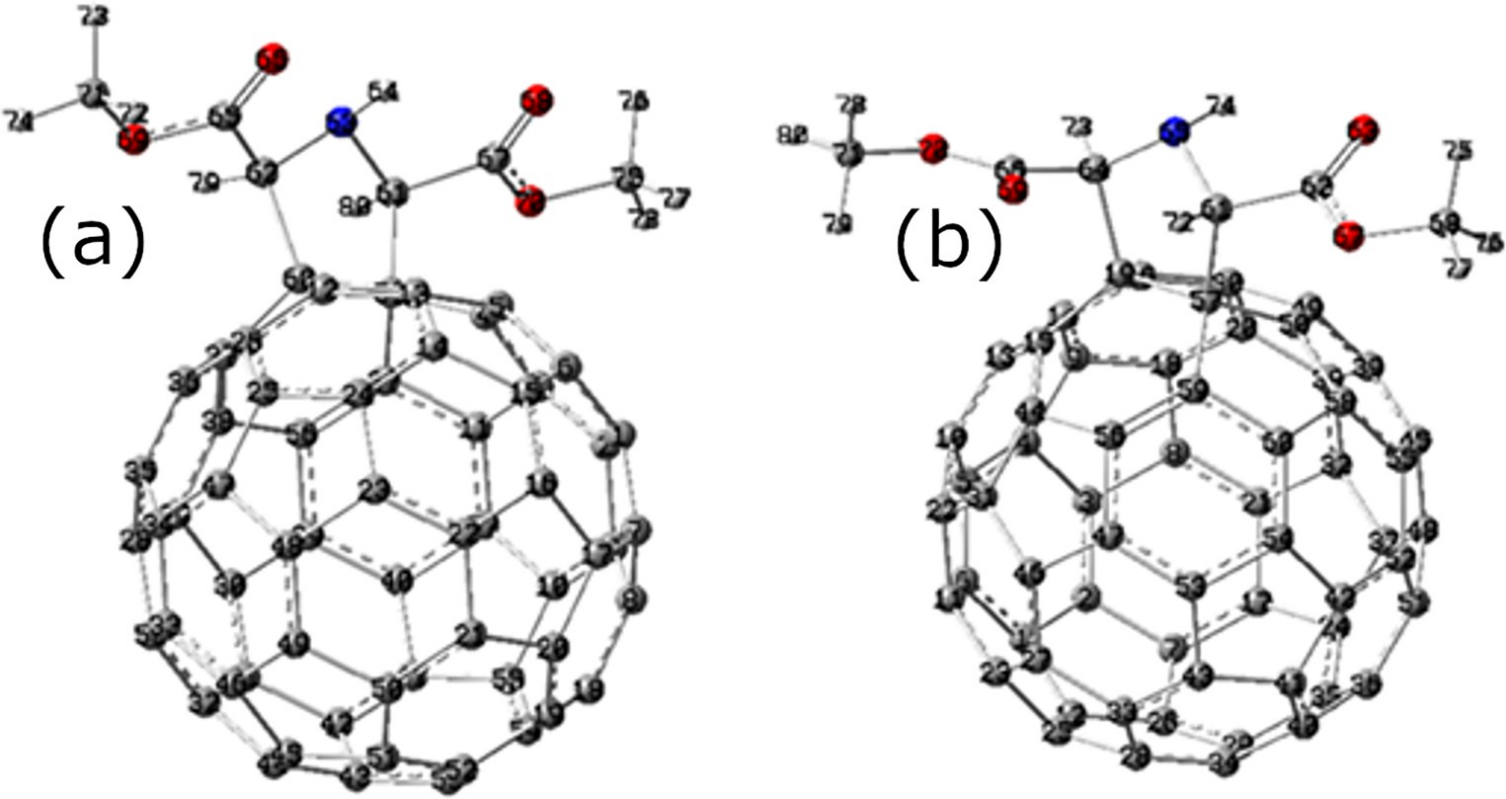
Representation of the (a) *cis* and (b) *trans* isomers of the fulleropyrrolidine mono-adduct.

We optimized the geometry of the two structures (*cis* and *trans*, S1 and S2 Tables) of the mono adduct by the DFT method at B3LYP 6–31 G(d) Level. The optimized geometry is represented in Fig 1.

The optimization of the two structures of the mono adduct (*cis* and *trans*) shows that the distances inside the two molecules are similar.

We were particularly interested in C-C bonding or grafting of the adduct. We note that the length values of these bounds are slightly shorter for the *trans* form compared to *cis* form (1.612 Å for C-C *Cis* versus 1.607 Å and 1.608 Å). This may suggest that adsorption energy for the *trans* form is the most important.

In the *cis* structure there is almost a Cs symmetry in the plane containing N and parallel to the two bonds between C_60_ and the Indole ring. *trans* structure is almost a C2 symmetry.

The calculated energies of the *cis* and *trans* isomers of the mono adduct in the gas state and in solution are presented in Table 1.

**Table 1:** Energy difference between the cis and *trans* isomers (kJ mol^-1^).

Structure	ΔE (gas phase)	ΔE (solvent CDCl_3_)
Mono-adduct (***cis***)	**0**	**0**
Mono-adduct (***trans***)	**4.07**	**5.25**

The formation of the *cis* form of the mono adduct is slightly more favorable than the formation of the *trans*, it’s worthy to note at room temperature, the difference between the two structures is about 1 kcal. This suggests that the proportions of the two molecules are almost equal. This result is in agreement with Maroto *et al*. who showed that *trans* isomer is less stable than *cis* for theses Fulleropyrrolidines [12]. The less favorable energy of the *trans* product could be mainly attributed to the higher repulsion of the lone pairs of the nitrogen atom of the pyrrolidine ring and the carbonyl group of the substituent.

### Calculation of IR spectra

**Fig 2:**
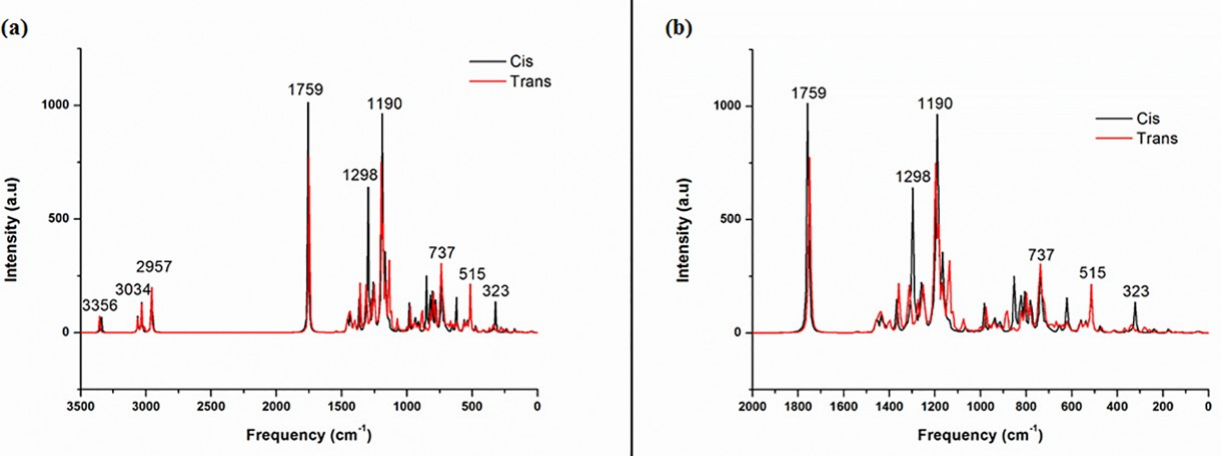
(a) Superposition of the two simulated IR full spectra of the *cis* and *trans* mono-adducts (b) Zoom in on the range from 0 to 2000 cm^-1^ of the same superposition.

The simulation of the IR spectra of the *cis* and *trans* isomers of the mono adduct is shown in Fig 2. The two spectra were superposed in order to identify the differences.

All characteristic frequencies of vibration assigned to the mono-adduct were determined and are summarized in Table 2.

**Table 2:** Comparison between experimental and calculated IR spectra for the *cis* and *trans* compounds with significant intensities (cm^-1^).

Attributions	*cis*	*trans*	EXP ^a^
-	323	-	-
-	737	737	-
-	783	783	-
-	806	-	-
-	852	-	-
-	-	883	-
-	-	1136	-
C-O	1167	1167	-
C-O	1190	1198	1255
C-H	1259	1251	-
C-H	1298	1313	-
C-N	1367	1359	1428
C = O	1756	1751	1740
N-H	3340	3356	3288

^**a**^ **According to Skanji et al** [5].

The comparison between the *cis* and the *trans* IR spectra (Table 2) shows a remarkable difference in the vibration frequency of 1298 cm^-1^. The latter is attributed to C-H bond, it has a higher intensity in the *cis* isomer IR spectrum. At the same time, this frequency value (1298 cm^-1^) is lower than the C-N frequency value that experimentally appears at 1428 cm^-1^. This is allows to suggest that we are in the presence of both *cis* and *trans* isomers [5].

### Calculation of chemical shifts

#### a) Calculation of ^1^H chemical shifts

For the optimized structures, the calculation of the ^1^H NMR spectra of the two isomers *cis* and *trans* of the mono adduct and their comparison with the experimental results is presented in Table 3.

**Table 3:** Comparison between calculated and experimental chemical shifts of the *cis* and *trans* isomers.

	*δ* (*ppm*) *cis*			RMSD	*δ* (*ppm*) *trans*			RMSD
	CH_3_	CH	NH		CH3	CH	NH	
**B3LYP/6-31g(d)**	4.01	5.24	4.15	**0.47**	3.98	5.86	3.65	**0.64**
**B3LYP/6-311+g (2d,p)**	4.11	5.97	4.68	**0.40**	4.14	6.5	4.31	**0.77 **
**EXP** ^a^	3.93	5.59	4.54		3.93	5.59	4.54	

^**a**^ **According to Skanji et al** [5]

Enlarging the basis allowed us the improvement of the shielding values. In addition to the 6–31 G(d) basis, we have used a larger basis, the 6–31 G (2d,p), to optimize the geometry and to simulate the NMR results [26].

Calculation of the shielding parameters was performed at the same level of theory as for the protons of the Tetra Methyl Silane (TMS) molecule, chosen as a reference, in order to obtain the chemical shifts for the protons and carbons under consideration through the following relation [27]:

δ=∂(TMS)−∂(Structure) (1)

Where, *δ* is the chemical shift in ppm.

The root mean square (rms) deviations between calculated and experimental values for the chemical shifts have been calculated using the following equation (27).

$$rms(P)=\sqrt{\frac{\sum_{K=1}^{N}{\left(P_{Exp}(K)-P_{Calc}(K)\right)}^{2}}{N}}$$ (2)

Where N is the number of data. These rms values calculated for *δ* are displayed in Table 3 and Table 4.

**Table 4:** Chemical shifts (ppm) calculated for the cis isomer in CDCl_3_.

	*δ* (*ppm*) *cis*			RMSD
	CH_3_	CH	NH	
**B3LYP/6-31g(d)**	4.01	5.24	4.15	0.47
**B3LYP/6-31g (d) + CDCl**_**3**_	**4.05**	**5.36**	**4.00**	0.51
**EXP** ^a^	3.93	5.59	4.54	

^**a**^**: According to Skanji et al.** [5].

We have considered that the nitrogen inversion in the Fulleropyrrolidines is a fast event at the operating temperatures, so we have calculated the average between the two chemical shifts of the two methine protons for the *trans* isomer [12].

We observe that for the *cis* isomer we have just one chemical shift at 5,24 ppm for the methine proton but for the *trans* isomer we have two chemical shifts at 5.34 and 6.39 ppm for the same proton we have averaged the two values in order to compare our results to the experimental results. Our system is non-symmetrical; we then have two different chemical shifts of the two methine protons. The deshielding of one of the protons from the other is due to the combination of the electronegative effect of nitrogen and the magnetic anisotropy created by the inhomogeneity of the electron density and the magnetic fields induced by the electron circulation within the fullerene cage [28].

Some studies with different experimental conditions have shown the protons in the *trans* form more deshielded than those in the *cis* form. In our case, we have shown the same trend [29,30]. For the basis 6-31G(d) The mean square error varies between 0.47 and 0.64 while for the basis 6–311+g (2d,p) it varies from 0.4 to 0.77. This suggests that hydrogen in CH *trans* is more deshielded than the proton of the CH *cis*.

#### b) Effect of solvent

We have studied the effect of the CDCl_3_ solvent which is used for the NMR analysis. The results of calculated NMR chemical shift for the most stable *cis* isomer of mono-adduct using the basis 6-31G(d) and the comparison with the results in gas phase are presented in Table 4.

The chemical shift for the *cis* structure has increased for the hydrogen of CH *cis*.

#### c) Calculation of ^13^C chemical shifts

**Fig 3:**
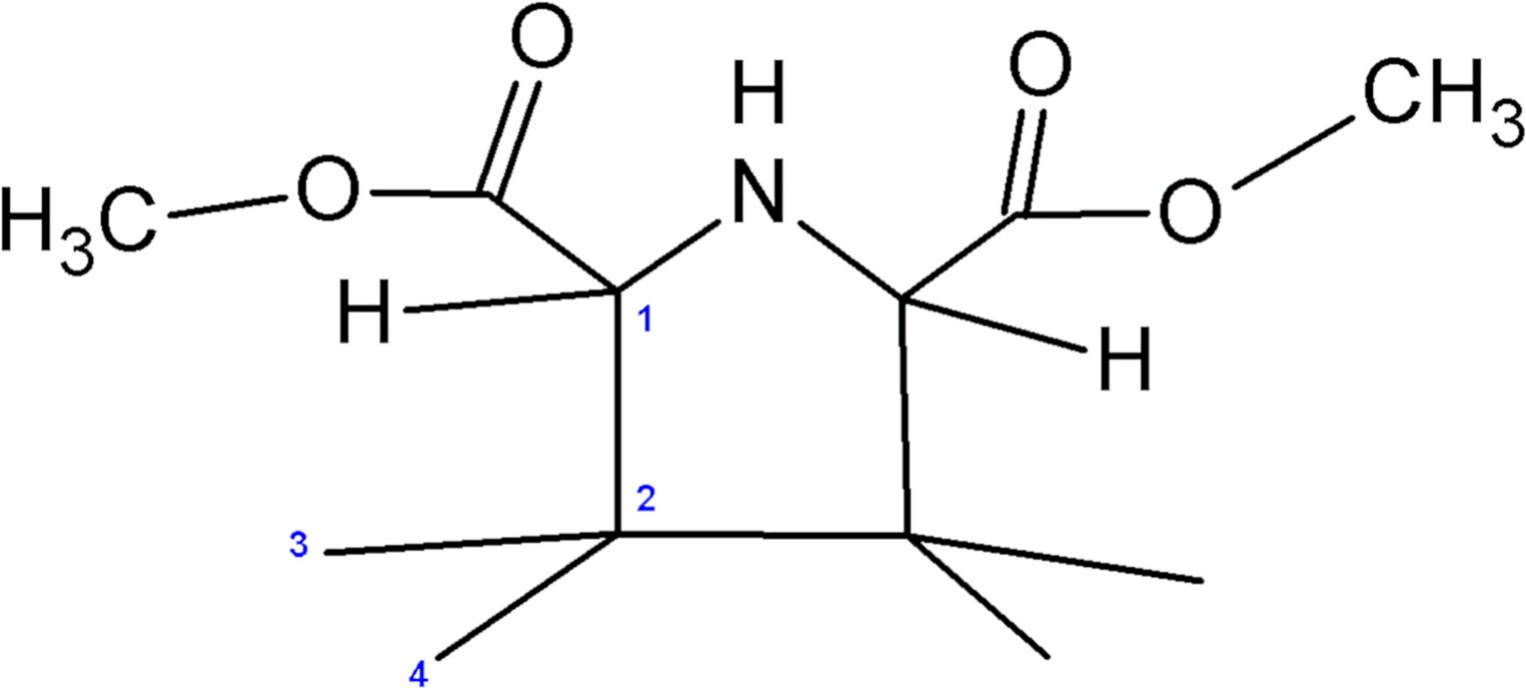
Different atoms of the adsorbed molecule.

The calculation of the predicted ^13^C chemical shifts in the gas phase, ^13^C of the characteristic groups (Fig 3), is illustrated in Table 5.

**Table 5:** Experimental and calculated ^13^C NMR spectra.

	C_1_	C_2_	C_3_	C_4_	Carboxylic Ester	CH_3_	RMSD
*δ* (*ppm*) EXP ^a^	74.0	-	149.84	152.1	160–170	52.9	-
*δ* (*ppm*) *cis*	**74.45**	**81.84**	**148.97**	**151.48**	**162.17**	**51.40**	**4.67**
*δ* (*ppm*) *trans*	**74.74**	**81.58**	**148.80**	**153.07**	**164.24**	**51.40**	**4.11**

^**a**^**: According to Skanji et al.** [5].

We have averaged the two values of ^13^C in order to compare our results to the experimental results.

In the ^13^C NMR experimental spectrum suggests that it is a *cis*—and *trans*- isomer mixture;

The Methine carbon is at 74 ppm, two fullerene sp2 carbons at 149,84 and 152.1 ppm, and Carboxylic Ester carbons between 160 and 170 ppm.

Considering the experimental results, the mean square error of the calculated NMR spectra of *cis* isomer is about 4.67 and is about 4.11 for the *trans* isomer.[5]

## Conclusion

Using B3LYP/6-31G(d), we have been able to model two configurations of the fulleropyrrolidine mono-adduct. We have notably shown that the *cis* isomer is more stable than the *trans* conformer. For the *cis* conformation, the simulated vibrational spectrum shows a more intense peak at 1298 cm^-1^. Also, the comparison with previously published experimental results has shown that the calculated ^1^H chemical shifts exhibit a significant difference between the two structures in both gas and solution phases. The trans isomer presents a proton at 5.86 ppm, which is more deshielded than the proton of the *cis* confomer (5.24 ppm). However ^13^C spectra has revealed no significant differences between the two isomers. These results will help rationalize the interpretation of the spectra of Fulleropyrrolidine structures.

## Supporting information

S1 Table XYZ coordinates of the optimized geometry: *cis*. (DOCX)

S2 Table XYZ coordinates of the optimized geometry: *trans*. (DOCX)

## References

[1] Goodarzi S, Da Ros T, Conde J, Sefat F, Mozafari M. Fullerene: biomedical engineers get to revisit an old friend. Vol. 20, Materials Today. 2017. p. 460–80.

[2] Moussa F. 5 –[60]Fullerene and derivatives for biomedical applications [Internet]. Nanobiomaterials. Elsevier Ltd.; 2018. 113–136 p. Available from: 10.1016/B978-0-08-100716-7.00005-2

[3] Martín N. New challenges in fullerene chemistry. Chem Commun [Internet]. 2006;(20):2093–104. Available from: http://xlink.rsc.org/?DOI=B601582B 10.1039/b601582b 16703124

[4] Lim SH, Cho DW, Choi J, An H, Shim JH, Mariano PS. SET-promoted photoaddition reactions of fullerene C_60_with tertiary N-trimethylsilylmethyl substituted α-aminonitriles. Approach to the synthesis of fulleropyrrolidine nitriles. Tetrahedron. 2017;73(44):6249–61.

[5] Skanji R, Ben Messaouda M, Zhang Y, Abderrabba M, Szwarc H, Moussa F. Sequential photo-addition of glycine methyl-ester to [60]fullerene. Tetrahedron. 2012;68(12):2713–8.

[6] De Person M, Coffre A, Skanji R, Ben Messaouda M, Abderraba M, Zhang Y, et al Mechanism and number of adducts of photo-addition of glycine methyl-ester to [60] fullerene. Tetrahedron [Internet]. Elsevier Ltd; 2013;69(33):6826–31. Available from: 10.1016/j.tet.2013.06.021

[7] Khemiri N, Messaoudi S, Moussa F, Abderrabba M, Chermette H. Theoretical investigation on two different mechanisms of fulleropyrrolidine formation. Theor Chem Acc. 2016;135(12).

[8] Gan L, Zhou D, Luo C, Tan H, Huang C, Lü M, et al Synthesis of fullerene amino acid derivatives by direct interaction of amino acid ester with C_60_. J Org Chem. 1996;61(6):1954–61.

[9] Vassilikogiannakis G, Orfanopoulos M. [2+2] Photocycloadditions of cis/trans-4-propenylanisole to C_60_. A step-wise mechanism. Tetrahedron Lett. 1997;38(24):4323–6.

[10] Nierengarten J, Oswald L, Nicoud J. Dynamic cis / trans isomerisation in a porphyrin–fullerene conjugate. 1998;1545–6.

[11] Chronakis N, Froudakis G, Orfanopoulos M. Stereochemistry of the [4 + 2 ] Cycloadditions of trans, trans- and cis, trans-2, 4-Hexadiene to C 60. Society. 2002;(13):3284–9. 10.1021/jo0109570 12003537

[12] Maroto EE, Mateos J, Garcia-Borràs M, Osuna S, Filippone S, Herranz MÁ, et al Enantiospecific cis-trans isomerization in chiral fulleropyrrolidines: Hydrogen-bonding assistance in the carbanion stabilization in H2O@C_60_. J Am Chem Soc. 2015;137(3):1190–7. 10.1021/ja5108854 25558918 10.1021/ja5108854

[13] Frisch MJ, Trucks GW, Schlegel HB, Scuseria GE, Robb MA, Cheeseman JR, et al. Gaussian 09, Revision A.02, Gaussian, Inc., Wallingford CT. Gaussian 09, Revision A.02, Gaussian, Inc., Wallingford CT 2009.

[14] Lee C, Hill C, Carolina N. into a functional of the electron density f f. 1988;37(2). 10.1103/physrevb.37.785 9944570

[15] Becke AD, Becke AD. Densityfunctional thermochemistry. III. The role of exact exchange Density-functional thermochemistry. III. The role of exact exchange. 1993;5648.

[16] Antony MP, Moehl T, Wielopolski M, Moser J. Long-Range p -Conjugation in Phenothiazine-containing Donor–Acceptor Dyes for Application in Dye-Sensitized Solar Cells. 2015;742:3859–68. 10.1002/cssc.201500931 26616683

[17] Hehre WJ, Ditchfield R, Pople JA. Self—Consistent Molecular Orbital Methods. XII. Further Extensions of Gaussian—Type Basis Sets for Use in Molecular Orbital Studies of Organic Molecules. 1972;2257(May 2012).

[18] September R. Commentationes The Influence of Polarization Functions on Molecular Orbital Hydrogenation Energies. 1973;28.

[19] Francl MM, Pietro WJ, Hehre WJ, Binkley JS, Gordon MS, Francl MM, et al Selfconsistent molecular orbital methods. XXIII. A polarizationtype basis set for secondrow elements Self-consistent molecular orbital methods. XXIII. A polarization-type basis set for second-row elements. 1982;3654.

[20] Krishnan R, Binkley JS, Seeger R, Pople JA, Krishnan R, Binkley JS, et al Selfconsistent molecular orbital methods. XX. A basis set for correlated wave functions Self-consistent molecular orbital methods. XX. A basis set for correlated wave functions. 1980;650.

[21] Mclean AD, Chandler GS. Contracted Gaussian basis sets for molecular calculations. I. Second row atoms, Z = 11–18 Contracted Gaussian basis sets for molecular calculations. I. Second row atoms, Z = 11–18. 1980;5639.

[22] Granadino-Roldán JM, Garzón A, Moral M, García G, Peña-Ruiz T, Paz Fernández-Liencres M, et al Theoretical estimation of the optical bandgap in a series of poly(aryl-ethynylene)s: A DFT study. J Chem Phys. 2014;140(4). 10.1063/1.4862802 25669584

[23] Chermette H. Density functional theory: A powerful tool for theoretical studies in coordination chemistry. Coord Chem Rev. 1998;

[24] Ditchfield R. Self-Consistent Perturbation-Theory of Diamagnetism. 1. Gauge-Invariant LCAO Method for NMR Chemical-Shifts. Mol Phys. 1974;27:789–807.

[25] Wolinski K, Hinton JF, Pulay P. Efficient Implementation of the Gauge-Independent Atomic Orbital Method for NMR Chemical Shift Calculations. J Am Chem Soc. 1990;112(23):8251–60.

[26] Tulyabaev AR, Kiryanov II, Samigullin IS, Khalilov LM. Are there reliable DFT approaches for 13C NMR chemical shift predictions of fullerene C_60_ derivatives? Int J Quantum Chem. 2017;117(1):7–14.

[27] Atieh Z, Allouche AR, Graveron-Demilly D, Aubert-Frécon M. Density functional theory (DFT) calculations of the proton nuclear magnetic resonance (NMR) spin-Hamiltonian parameters for serine. Meas Sci Technol. 2011;22(11). 10.1088/0957-0233/27/11/115201

[28] Klod S, Kleinpeter E. Ab initio calculation of the anisotropy effect of multiple bonds and the ring current effect of arenes—application in conformational and configurational analysis. J Chem Soc Perkin Trans 2 [Internet]. 2001;(10):1893–8. Available from: http://xlink.rsc.org/?DOI=b009809o

[29] Wei F, Furihata K, Hu F, Miyakawa T. Complex mixture analysis of organic compounds in green coffee bean extract by two‐dimensional NMR spectroscopy. Magn Reson [Internet]. 2010;48:857–65. Available from: http://onlinelibrary.wiley.com/doi/10.1002/mrc.2678/full 10.1002/mrc.2678 20818806

[30] Kashif A, Federica M, Eva Z, Martina R, Young HC, Robert V. NMR metabolic fingerprinting based identification of grapevine metabolites associated with downy mildew resistance. J Agric Food Chem. 2009;57(20):9599–606. 10.1021/jf902069f 19785416 10.1021/jf902069f

