# Supplementary material for: Density Functional Theory based study on structural, vibrational and NMR properties of cis - trans fulleropyrrolidine mono-adducts
Source: PLoS One. 2018 Nov 19;13(11):e0207635. doi: 10.1371/journal.pone.0207635 (PMC6242360; doi:10.1371/journal.pone.0207635)
Supplement: S1 Table — (DOCX) [file pone.0207635.s001.docx]

**S1 Table. XYZ coordinates of the optimized geometry: *cis*.**

CIS

C 0.04417300 2.31216600 2.48414300

C -0.24261700 3.03266300 1.26773600

C -1.26438200 2.57817000 0.42740300

C -2.06601900 1.43374700 0.77479500

C -1.77923800 0.73878600 1.92903600

C -0.70810600 1.17559200 2.79833200

C 1.48247300 2.31049900 2.67895500

C 2.08569600 3.03852200 1.57410400

C 1.01687500 3.48754000 0.69913500

C 1.19891900 3.48840200 -0.68283900

C -1.07489100 2.58076700 -1.01069000

C -2.06591100 -1.43392600 0.77468500

C -1.77918400 -0.73903500 1.92897800

C -0.70803000 -1.17583700 2.79824200

C -0.04788700 -0.00012200 3.33599800

C 1.33278100 -0.00008600 3.53661500

C 2.11509900 1.17721000 3.19419500

C 3.29569400 2.60361700 1.02942200

C 3.95251400 1.42569100 1.56813300

C 3.37313200 0.72785900 2.63009000

C 3.37317800 -0.72783100 2.63003500

C 2.11517500 -1.17730500 3.19410600

C 1.48262100 -2.31059800 2.67878100

C 0.04432200 -2.31234200 2.48397000

C -0.24242200 -3.03276700 1.26750900

C -1.26421700 -2.57827800 0.42721100

C -1.75814000 1.43818300 -1.55321900

C 0.72054300 -2.31295600 -2.65901600

C 0.07489700 -1.17636900 -3.15805300

C 0.85170700 0.00016700 -3.50502400

C 2.23731100 0.00020400 -3.34189900

C 2.90439200 -1.17673300 -2.80834400

C 2.45773600 -3.03849900 -1.25185500

C 1.19914200 -3.48826300 -0.68310200

C 0.12976900 -3.03413200 -1.55791800

C -1.07472400 -2.58075200 -1.01088300

C -1.75803500 -1.43816200 -1.55331900

C -1.18510100 -0.73988000 -2.59474700

C 0.07482000 1.17662500 -3.15796400

C 0.72039600 2.31321800 -2.65884000

C 2.16022600 2.31058000 -2.47475600

C 2.90431700 1.17714100 -2.80825500

C 3.97409400 0.72803300 -1.93858900

C 3.97414000 -0.72762300 -1.93864400

C 4.25919600 -1.42565200 -0.76303900

C 3.48509000 -2.60320100 -0.41248500

C 1.01709800 -3.48751900 0.69887200

C 2.08589100 -3.03849800 1.57387500

C 3.29586200 -2.60347600 1.02922600

C 3.95260500 -1.42554700 1.56802600

C 4.54942700 -0.69771500 0.46081700

C 4.54938400 0.69798100 0.46087000

C 4.25910500 1.42599100 -0.76293100

C 3.48492500 2.60346700 -0.41228800

C 2.45754400 3.03876100 -1.25162500

C -1.18515300 0.74001500 -2.59469500

C 0.12957600 3.03426700 -1.55769000

C 2.16037300 -2.31024300 -2.47493100

C -2.65673700 0.80319700 -0.49186100

C -2.65666700 -0.80332200 -0.49191700

C -4.21478900 1.15148200 -0.71464000

C -4.21467200 -1.15174500 -0.71473200

N -4.98270000 -0.00018300 -0.31842500

H -5.16297800 -0.00021800 0.68666600

C -4.66162000 -2.38094700 0.05884000

O -5.19204500 -2.34250800 1.14675800

C -4.66187700 2.38060100 0.05899600

O -5.19170600 2.34208900 1.14720100

O -4.36226000 -3.50835600 -0.60809600

O -4.36336000 3.50804900 -0.60825100

C -4.67894600 -4.73649100 0.07647300

H -4.12964900 -4.79699800 1.01911100

H -5.75107400 -4.79127100 0.27945900

H -4.37215700 -5.53301600 -0.60097100

C -4.68018400 4.73614200 0.07633000

H -5.75218600 4.79038800 0.28011900

H -4.13020400 4.79711300 1.01854100

H -4.37432600 5.53270600 -0.60148800

H -4.34470000 -1.33860800 -1.78616100

H -4.34486100 1.33837900 -1.78605700
