# Supplementary material for: Density Functional Theory based study on structural, vibrational and NMR properties of cis - trans fulleropyrrolidine mono-adducts
Source: PLoS One. 2018 Nov 19;13(11):e0207635. doi: 10.1371/journal.pone.0207635 (PMC6242360; doi:10.1371/journal.pone.0207635)
Supplement: S2 Table — (DOCX) [file pone.0207635.s002.docx]

**S2 Table. XYZ coordinates of the optimized geometry: *trans*.**

TRANS

C -3.54442600 2.59209500 -0.22316500

C -3.63016000 1.98830700 -1.54318100

C -2.57572800 2.13341300 -2.44684900

C -1.39336500 2.88612700 -2.06605500

C -1.31125600 3.46521900 -0.80030200

C -2.40813400 3.31726400 0.14037700

C -4.29811900 0.70602200 -1.40720700

C -2.14524300 0.99999500 -3.24999800

C -0.23675200 2.21167900 -2.63425600

C -0.06929500 3.39657900 -0.04704900

C -1.83966500 3.15942800 1.46945600

C -4.15963400 1.68240000 0.72699600

C 1.03732700 2.73232700 -0.58562300

C 0.95181700 2.12862600 -1.90133500

C 1.88395500 1.90650100 0.23221700

C 1.74349100 0.92698600 -1.89612000

C -3.88553200 -0.38179500 -2.17949800

C -0.69933300 1.05542400 -3.36377100

C 2.60566700 0.86754800 -0.63107900

C 0.05307400 -0.12333800 -3.32496100

C -2.78789900 -0.23245600 -3.11537600

C -0.39422400 3.20976300 1.34652100

C -2.43152000 2.28200400 2.38021200

C -3.79041600 -1.70515900 -1.58021300

C -3.61422200 1.53219400 2.00374300

C -4.62706400 0.51711000 -0.00460100

C -1.60074200 1.42087700 3.20711900

C -3.51907600 0.20895700 2.60287100

C -4.53633300 -0.75161500 0.57010500

C 1.29506200 -0.17927100 -2.58347200

C 0.40581200 2.37056200 2.12947400

C -2.00886200 -1.45993400 -3.09073100

C -2.27804700 0.14206200 3.34959900

C -3.97360900 -0.90925000 1.90074600

C -4.11224900 -1.88583200 -0.23330000

C -3.29034800 -2.74489600 0.60084800

C -2.63371000 -2.37211600 -2.14623500

C -0.61803700 -1.40322200 -3.18691300

C 0.20657100 -2.26108000 -2.35506800

C -3.20431900 -2.14082300 1.92073800

C -2.01106700 -2.20700200 2.64311300

C -0.85512300 -2.87616200 2.07246300

C -1.53715600 -1.04129800 3.37263400

C 1.56350400 1.71506700 1.55896500

C -0.39693500 -3.14864900 -1.45784800

C -0.21224600 1.46158600 3.07718400

C 0.56152300 0.23323800 3.09831800

C -1.84308800 -3.20028400 -1.34692100

C 1.39088800 -1.52280700 -1.97369300

C 1.93124200 -1.67692700 -0.71630200

C -2.17903500 -3.39080100 0.05547300

C -0.93767800 -3.45507200 0.80685400

C -0.09106800 -0.99472900 3.25185300

C 0.32792800 -2.11980400 2.45200700

C 0.15939800 -3.30150000 -0.13574300

C 1.66325400 0.37109600 2.16954300

C 2.71361600 -0.59039200 0.03240500

C 1.28890300 -2.55803900 0.22258500

C 2.07680300 -0.70818800 1.41785100

C 1.37630000 -1.95827000 1.54040800

C 4.30049900 -0.85404300 0.04179400

C 4.65788400 -2.30409300 -0.23213600

O 4.95166500 -2.73394100 -1.32735100

C 4.13352300 1.23224800 -0.97211600

N 4.87923500 -0.00680900 -0.97949600

C 4.69617900 2.18313800 0.08471900

O 4.57819100 -3.04694800 0.88290300

C 4.83390800 -4.45459900 0.71114700

O 5.09448300 1.85393900 1.18058300

O 4.65755700 3.45514600 -0.35401200

C 5.09408400 4.44900000 0.59268600

H 4.67526500 -0.55771900 1.02264800

H 4.19555100 1.72552800 -1.94306100

H 4.81632700 -0.47920200 -1.88082400

H 5.84175000 -4.61124200 0.31952600

H 4.10666700 -4.89016500 0.02152300

H 4.73296600 -4.89059800 1.70470700

H 6.13378700 4.27120900 0.87778000

H 4.46653900 4.42119300 1.48704100

H 4.99237600 5.40471200 0.07872000
